# Early pregnancy metabolic factors associated with gestational diabetes mellitus in normal-weight women with polycystic ovary syndrome: a two-phase cohort study

**DOI:** 10.1186/s13098-019-0462-6

**Published:** 2019-08-23

**Authors:** Wei Zheng, Wenyu Huang, Li Zhang, Zhihong Tian, Qi Yan, Teng Wang, Lirui Zhang, Guanghui Li

**Affiliations:** 1 Division of Endocrinology and Metabolism, Department of Obstetrics, Beijing Obstetrics and Gynecology Hospital, Capital Medical University, No 251, Yaojiayuan Road, Chaoyang District, Beijing, 100026 China; 2 Division of Endocrinology, Metabolism and Molecular Medicine, Northwestern University Feinberg School of Medicine, Chicago, IL 60611 USA

**Keywords:** Polycystic ovary syndrome, Metabolic indicators, Gestational diabetes, Insulin resistance, Body mass index

## Abstract

**Background:**

Polycystic ovary syndrome (PCOS) has been consistently associated with subsequent gestational diabetes mellitus (GDM). Women with PCOS showed a high prevalence of obesity, which raises the question regarding the role of obesity or PCOS *pe ser* in development of GDM. In this study we conducted a 2-phase study to compare the risk of GDM and its associated early pregnancy metabolic factors in women with and without PCOS, stratified by pre-pregnancy body mass index (BMI).

**Methods:**

A 2-phase design was used in this study. The initial phase of the study included 566 age- and pre-pregnancy BMI-matched singleton pregnant women (242 with and 324 without PCOS). Risk of GDM and associated early-pregnancy risk factors were explored between women with and without PCOS, after stratification by pre-pregnancy BMI. Stratified analysis was conducted in normal weight (pre-pregnancy BMI < 25 kg/m^2^) and overweight/obese (pre-pregnancy BMI ≥ 25 kg/m^2^) groups. Subsequently, the findings was confirmed in a separate cohort study with 18,106 participants (877 with and 17,229 without PCOS).

**Results:**

Overall, prevalence of GDM is higher in women with PCOS. Results from the initial study showed that in normal-weight subjects, there is a significant increase in GDM prevalence in PCOS women than non-PCOS women (26.5% vs. 16.2%, p = 0.02). Additionally, normal-weight PCOS women showed higher triglycerides levels (1.51 ± 0.84 mmol/L vs. 1.30 ± 0.75 mmol/L, p = 0.02), lower SHBG levels (277.8 ± 110.2 nmol/L vs. 330.5 ± 180.4 nmol/L, p = 0.001) and a possible trend towards higher insulin resistance (LogHoMA-IR 0.70 ± 0.55 vs. 0.57 ± 0.57, p = 0.05) during early pregnancy. However, in overweight/obese group, no difference in risk of GDM was observed between PCOS and non-PCOS subjects (p = 0.7). Results from the independent cohort confirmed the risk for GDM associated with PCOS in normal weight women (p < 0.0001).

**Conclusion:**

Consistent findings from the 2-phase study showed an increased risk of GDM in normal-weight, but not overweight/obese PCOS women. Analysis of early-pregnancy risk factors of GDM suggested that the pathogenesis of GDM in normal weight and overweight/obese women with PCOS may be different.

## Introduction

Polycystic ovary syndrome (PCOS) is the most common reproductive disorder in women of childbearing age. Hyperandrogenaemia, chronic anovulation, and polycystic ovaries were three significant characteristic features of PCOS [1]. Additionally, PCOS is associated with insulin resistance and increased risk for metabolic disease, especially diabetes [2–4]. A recent meta-analysis by Boomsma et al. demonstrated that PCOS is associated with increased risk of adverse pregnancy outcomes including gestational diabetes (GDM), pregnancy-induced hypertension (PIH), pre-eclampsia, preterm birth, as well as elevated perinatal mortality [5]. Accumulating evidence indicates that the increased risk of GDM in PCOS subjects is likely due to higher insulin resistance before pregnancy [6–9]. Overweight and obesity is highly prevalent in PCOS and has been associated with insulin resistance and subsequent diabetes. However, the role of obesity in the development of GDM in women with PCOS remains controversial [4, 6, 9]. Previous studies have shown that the level of insulin resistance in women with PCOS differ between groups with different body mass index (BMI) [10, 11]. A large cohort study by Ollila et al. demonstrated impaired glucose metabolism in overweight and obese but not normal weight women with PCOS [4]. However, a recent meta-analysis reported that PCOS per se increases risk for GDM independent of BMI [9]. Herein, we conducted a two-phase cohort study to compare the risk of GDM and its associated early pregnancy metabolic factors in women with and without PCOS in different weight groups.

## Materials and methods

### Study design and participants

This initial cohort study included 242 women with PCOS and 324 age- and pre-pregnancy BMI (PPBMI)-matched healthy controls recruited from January 2013 to December 2015 in Beijing, China. Participants were recruited at gestational week 8–15. Women with singleton pregnancy, 18–45 years of age, a history of PCOS (or age and PPBMI-matched healthy controls) were enrolled in the study. Participants with pre-existing chronic disease including diabetes, hypertension, liver, kidney, thyroid or cardiovascular disease were excluded. PCOS was diagnosed before conception according to the modified Rotterdam Criteria. For each participants with PCOS, we randomly selected one or two age- and PPBMI-matched controls using an early pregnancy registration system of the hospital.

In a separate cohort, data from 877 PCOS and 17,229 non-PCOS women who delivered from February 2016 and December 2017 were used to confirm findings from the first cohort. The distinct cohort study included all eligible 18–45 years old singleton pregnancy women without pre-existing chronic disease as mentioned above. The study was approved by the Ethics Committee of Beijing Obstetrics and Gynecology Hospital (2012-KY-012, 2016-KY-066). Written informed consent was obtained from all participants. All procedures were performed in compliance with the Declaration of Helsinki.

### Early pregnancy metabolic characteristics

Baseline information were collected through review of medical records. Pre-pregnancy weight was self-reported. The documented weight at the patient’s first visit to the hospital (5–6 weeks of gestation) was used when the participants could not remember their pre-pregnancy weight. Women were classified to be normal weight (pre-pregnancy BMI < 25 kg/m^2^) and overweight/obese (pre-pregnancy BMI ≥ 25 kg/m^2^) according to pre-pregnancy BMI. Fasting blood samples were collected during the first prenatal visit at gestational week 8–15. Fasting plasma glucose (FPG) was determined by hexokinase method using ARCHITECT GLUCOSE (3L82-21) kit following the standard protocol with an automatic biochemical analyzer (ARCHITECT ci16200 analyzer, Abbott Laboratories, IL, USA). Fasting lipid profiles including total cholesterol (TC), triglyceride (TG), high density lipoprotein cholesterol (HDL-C), low density lipoprotein cholesterol (LDL-C) and lipoprotein a (Lp (a)) were measure by enzymatic method using ARCHITECT CHOLESTOROL (7D62-21), TRIGLYCERIDE (7D74-21), ULTRA HDL (3K33-21), DIRECT LDL (1E33-20), and QUANTIA LP (a) (7K00-01) (ARCHITECT ci16200 analyzer, Abbott Laboratories, IL, USA). Additionally, fasting insulin (FINS), adiponectin (APN) and sex hormone-binding globulin (SHBG) levels were assayed using Human/Canine/Porcine Insulin Quantikine ELISA Kit (DINS00), Human Total Adiponectin/Acrp30 Quantikine ELISA Kit (DRP300), and Human SHBG Quantikine ELISA Kit (DSHBG0B) following a standard protocol (R&D Systems, Minneapolis, MN, USA). Insulin resistance (IR) was assessed using following indicators: Homeostatic model assessment-insulin resistance score (HOMA-IR = FINS × FPG/22.5) and quantitative insulin sensitivity check index (QUICKI = 1/ (Log (FINS) + Log (GLU*18)).

### Pregnancy outcomes

Subjects were followed every 3 months until delivery. GDM was diagnosed according to the 2013 American Diabetes Association (ADA) criteria. A 75-g oral glucose tolerance test was performed at gestational week 24–28. The participants were diagnosed with GDM if any one of following criteria of plasma glucose was met: fasting ≥ 5.1 mmol/L, 1 h ≥ 10.0 mmol/L, or 2 h ≥ 8.5 mmol/L. PIH was defined as new onset of elevated blood pressure (systolic blood pressure ≥ 140 mmHg or diastolic blood pressure ≥ 90 mmHg) in a previously normotensive woman. PIH with one of following features was considered pre-eclampsia: Proteinuria of > 300 mg/24 h, proteinuria/creatinine ≥ 0.3 or new onset of related organ dysfunction [12]. Birth weight and gestational weeks were collected from medical record. Low birth weight (LBW) and macrosomia is defined as neonatal birth weight < 2500 g and > 4000 g, respectively. Large for gestational age (birth weight above the 90th percentile for gestational age) and small for gestational age (birth weight below the 10th percentile for gestational age) were defined by the international standards [13]. Gestational age < 37 week is considered as preterm.

### Confirmation of finding in a separate cohort

We then conducted analysis in a separate cohort, composed of PCOS and non-PCOS women who delivered from February 2016 and December 2017, to confirm our findings from the first cohort. We examined the prevalence of PCOS in the new cohort. Then we evaluated risk for GDM in PCOS and non-PCOS women stratified by PPBMI.

### Statistical analysis

Data was analyzed using SAS 9.3. We first used student *t* test or Chi square test to compare basic characteristics of the PCOS and non-PCOS women based on the types of variables. We transformed HOMA-IR to Log^HoMA−IR^ since HOMA-IR level do not exhibit normal distribution. Then we examined the association between PCOS and pregnancy outcomes stratified by PPBMI. Pairwise comparison in women with different PCOS phenotype and PPBMI was made using Tukey method. Subsequently, we compared early pregnancy risk factors of GDM in women with and without PCOS stratified by PPBMI using student t-test for numeric variables and Chi square test/fisher’s exact test for categorical variables. At last, we confirmed the risk for GDM associated with PCOS in normal weight and overweight/obese women using data from a separate cohort. Any p < 0.05 was considered statistically significant, and all statistical tests were performed with a two-side examination.

## Results

A total of 566 participants were included in the initial cohort, including 242 women with PCOS and 324 age- and PPBMI-matched non-PCOS controls (Table 1). PCOS subjects, as compared to non-PCOS controls, have higher rate of in vitro fertilization and embryo transfer (IVF–EF), lower parity and similar prevalence of positive family history of diabetes and hypertension.

**Table 1:** Basic characteristics of pregnant women with and without PCOS in the initial cohort [mean ± SD or n (%)]

	PCOS (n = 242)	Non-PCOS (n = 324)	p-value*
Age, years, mean ± SD	30.32 ± 3.02	30.56 ± 3.44	0.3
Gravidity, mean ± SD	1.60 ± 0.93	1.81 ± 1.03	*0.01*
Parity, mean ± SD	1.05 ± 0.23	1.06 ± 0.25	0.7
History of EPL, n (%)	52 (21.49)	67 (20.68)	0.8
IVF–ET, n (%)	26 (10.74)	9 (2.78)	< *0.0001*
PPBMI, kg/m^2^, mean ± SD	24.37 ± 4.36	24.52 ± 5.00	0.4
Family history of DM, n (%)	50 (20.66)	51 (15.74)	0.1
Family history of hypertension, n (%)	68 (28.10)	77 (23.77)	0.2

Italic values are statistically significant at p < 0.05

Family history of DM and hypertension means related history of first degree relatives

*PCOS* polycystic ovary syndrome, *EPL* early pregnancy loss, *IVF–ET* in vitro fertilization and embryo transfer, *PPBMI* pre-pregnancy body mass index, *DM* diabetes mellitus

* p-value was calculated by student t-test for numeric variables and by Chi square test for categorical variables

**Fig. 1:**
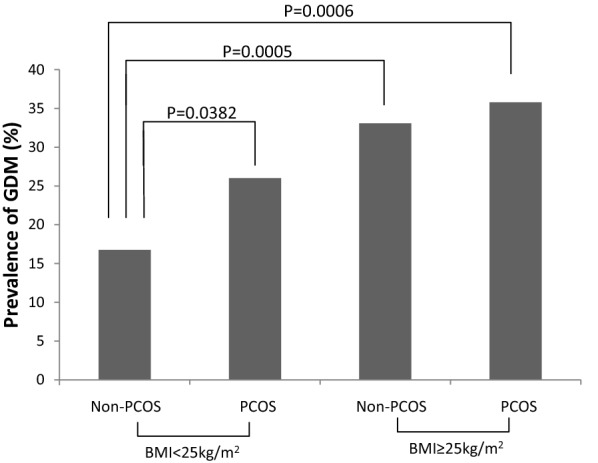
Prevalence of GDM in normal weight and overweight/obese women with and without PCOS (significant differences for pairwise comparison using Tukey method were indicated)

PCOS associated pregnancy outcomes were shown in Table 2 and Fig. 1. Overall, PCOS is associated with nearly significant higher prevalence of GDM, preterm and lower birthweight while no significant difference in other pregnancy outcomes between PCOS and non-PCOS subjects was observed (Table 2). We further stratified the subjects by their pre-pregnancy body weight into two groups: normal weight and overweight/obesity (Table 2). In normal weight women, PCOS is associated with a significant increase in GDM incidence (26.5% vs. 16.1%, p = 0.02). In contrast to the findings in the normal weight group, no significant difference in GDM incidence was found in the overweight/obesity group (35.8% vs. 33.1%, p = 0.7). There is no statistical difference in the prevalence of GDM between normal weight PCOS subjects vs. overweight/obese subjects (with or without PCOS) (p = 0.19 and p = 0.11, respectively) in the initial cohort (Fig. 1). However, related significant differences were observed in the confirmative study (normal weight women with PCOS vs. overweight/obese women without PCOS: p < 0.0001; normal weight women with PCOS vs. overweight/obese women with PCOS: p = 0.0008).

**Table 2:** Pregnancy outcomes in women with and without PCOS stratified by PPBMI in the initial cohort

	PPBMI	PCOS (n = 242)	Non-PCOS (n = 324)	p-value*
GDM, n (%)	< 25 kg/m^2^	39 (26.5)	30 (16.2)	*0.02*
	≥ 25 kg/m^2^	34 (35.8)	46 (33.1)	0.7
	Total	73 (30.2)	76 (23.5)	0.07
PIH + Pre-eclampsia, n (%)	< 25 kg/m^2^	9 (6.1)	12 (6.5)	0.9
	≥ 25 kg/m^2^	21 (22.1)	30 (21.6)	0.9
	Total	30 (12.4)	42 (12.96)	0.8
PIH, n (%)	< 25 kg/m^2^	6 (4.1)	7 (3.8)	0.9
	≥ 25 kg/m^2^	14 (14.7)	19 (13.7)	0.8
	Total	20 (8.3)	26 (8.0)	0.8
Pre-eclampsia, n (%)	< 25 kg/m^2^	3 (2.0)	5 (2.7)	0.9
	≥ 25 kg/m^2^	7 (7.4)	11 (7.9)	0.9
	Total	10 (4.1)	16 (4.9)	0.7
Delivery week, weeks, mean (SD)	< 25 kg/m^2^	38.8 (1.2)	38.8 (1.3)	0.6
	≥ 25 kg/m^2^	38.2 (2.1)	38.5 (1.4)	0.2
	Total	38.5 (1.7)	38.7 (1.4)	0.2
Preterm delivery, n (%)	< 25 kg/m^2^	4 (2.7)	1 (0.5)	0.1
	≥ 25 kg/m^2^	8 (8.4)	4 (2.9)	0.07
	Total	12 (5.0)	5 (1.5)	*0.02*
PROM, n (%)	< 25 kg/m^2^	42 (28.6)	44 (23.8)	0.3
	≥ 25 kg/m^2^	19 (20.0)	34 (24.5)	0.4
	Total	61 (25.2)	78 (24.1)	0.8
Birth weight, g, mean (SD)	< 25 kg/m^2^	3379.7 (507.7)	3454.8 (442.7)	0.2
	≥ 25 kg/m^2^	3407.6 (602.2)	3521.4 (503.8)	0.1
	Total	3390.6 (545.7)	3483.8 (407.3)	*0.03*
LGA, n (%)	< 25 kg/m^2^	33 (22.5)	36 (19.5)	0.5
	≥ 25 kg/m^2^	28 (29.5)	54 (38.9)	0.14
	Total	61 (25.2)	90 (27.8)	0.5
Macrosomia, n (%)	< 25 kg/m^2^	11 (7.5)	16 (8.7)	0.7
	≥ 25 kg/m^2^	13 (13.7)	20 (14.4)	0.9
	Total	24 (9.9)	36 (11.1)	0.6
SGA, n (%)	< 25 kg/m^2^	4 (2.7)	2 (1.1)	0.18
	≥ 25 kg/m^2^	0 (0.0)	5 (3.6)	0.08
	Total	4 (1.7)	7 (2.2)	0.7
Low birth weight, n (%)	< 25 kg/m^2^	6 (4.1)	4 (2.2)	0.3
	≥ 25 kg/m^2^	5 (5.3)	7 (5.0)	0.9
	Total	11 (4.6)	11 (3.4)	0.5
Congenital anomaly, n (%)	< 25 kg/m^2^	1 (0.7)	2 (1.1)	1
	≥ 25 kg/m^2^	2 (2.1)	0	0.2
	Total	3 (1.2)	2 (0.6)	0.7

Italic values are statistically significant at p < 0.05

*PCOS* polycystic ovary syndrome, *PPBMI* pre-pregnancy body mass index, *GDM* gestational diabetes, *PIH* pregnancy induced hypertension, *PROM* premature rupture membrane, *LGA* large for gestational age, *SGA* small for gestational age, *LBW* low birth weight

* p-value was calculated by student t-test for numeric variables and by Chi square test for categorical variables

We subsequently evaluated a number of potential early-pregnancy risk factors for GDM (Table 3 and Table 4). In normal weight subgroup, PCOS subjects were found to have a possible trend toward higher insulin resistance (fasting insulin 10.77 ± 5.45 mIU/L vs. 9.62 ± 4.89 mIU/L, p = 0.06, Cohen’s d = 0.22; Log^HoMA−IR^ 0.70 ± 0.55 vs. 0.57 ± 0.57, p = 0.05, Cohen’s d = 0.23; QUICKI 0.34 ± 0.03 vs. 0.35 ± 0.03, p = 0.05, Cohen’s d = 0.23), higher triglyceride (1.51 ± 0.84 mmol/L vs. 1.30 ± 0.75 mmol/L, p = 0.02), lower SHBG (277.8 ± 110.2 nmol/L vs. 330.5 ± 180.4 nmol/L, p = 0.001) and LDL-C (1.97 ± 0.50 mmol/L vs. 2.13 ± 0.52 mmol/L, p = 0.004), and lower blood pressures (SBP, 110.6 ± 11.7 mmHg vs. 114.2 ± 11.4 mmHg, p = 0.006; DBP, 69.4 ± 9.3 mmHg vs. 71.9 ± 9.0 mmHg, p = 0.01) (Table 3).

**Table 3:** Comparison of early pregnancy factors for GDM in normal weight women (PPBMI < 25 kg/m^2^) with and without PCOS

	PCOS (n = 147)	Non-PCOS (n = 185)	p-value*
Gravidity, mean ± SD	1.52 ± 1.38	1.70 ± 1.56	0.08
Parity, mean ± SD	1.04 ± 1.01	1.06 ± 1.03	0.4
History of EPL, n (%)	33 (22.5)	33 (17.8)	0.3
IVF–ET, n (%)	16 (10.9)	5 (2.7)	*0.002*
Family history of DM, n (%)	24 (16.3)	23 (12.4)	0.3
Family history of hypertension, n (%)	33 (22.5)	28 (15.1)	0.09
SBP, mmHg, mean ± SD	110.6 ± 11.7	114.0 ± 11.4	*0.006*
DBP, mmHg, mean ± SD	69.4 ± 9.3	71.9 ± 9.0	*0.01*
FPG, mmol/L, mean ± SD	4.83 ± 0.53	4.74 ± 0.49	0.1
Fins, mIU/L, mean ± SD	10.77 ± 5.45	9.62 ± 4.89	0.06
Log^HoMA−IR^, mean ± SD	0.70 ± 0.55	0.57 ± 0.57	0.05
QUICKY, mean ± SD	0.34 ± 0.03	0.35 ± 0.03	0.05
Lipid profile			
TC, mmol/L, mean ± SD	4.50 ± 0.73	4.41 ± 0.76	0.3
TG, mmol/L, mean ± SD	1.51 ± 0.84	1.30 ± 0.75	*0.02*
HDL, mmol/L, mean ± SD	1.93 ± 1.60	1.98 ± 0.73	0.7
LDL, mmol/L, mean ± SD	1.97 ± 0.50	2.13 ± 0.52	*0.004*
ApoA1, mmol/L, mean ± SD	1.53 ± 0.37	1.47 ± 0.29	0.06
ApoB, mmol/L, mean ± SD	0.69 ± 0.15	0.72 ± 0.15	0.06
Lp (a), mg/L, mean ± SD	252.4 ± 296.3	286.5 ± 283.5	0.3
APN, μg/mL, mean ± SD	75.25 ± 43.59	72.61 ± 36.26	0.6
SHBG, nmol/L, mean ± SD	277.8 ± 110.2	330.5 ± 180.4	*0.001*

Italic values are statistically significant at p < 0.05

Family history of DM and hypertension means related history of first degree relatives

*GDM* gestational diabetes, *PPBMI* pre-pregnancy body mass index, *PCOS* polycystic ovary syndrome, *EPL* early pregnancy loss, *IVF–ET* in vitro fertilization and embryo transfer, *DM* diabetes mellitus, *SBP* systolic blood pressure, *DBP* diastolic blood pressure, *FPG* fasting plasma glucose, *Fins* fasting insulin, *HoMA-IR* Homeostasis Model Assessment-insulin Resistance, *QUICKI* quantitative insulin sensitivity check index, *TC* total cholesterol, *TG* triglycerides, *HDL* high density lipoprotein-cholesterol, LDL low density lipoprotein-cholesterol, *ApoA1* apolipoprotein A-I, *ApoB* apolipoprotein B, *Lp (a)* lipoprotein a, *APN* adiponectin, *SHBG* sex hormone-binding globulin

* p-value was calculated by student t-test for numeric variables and by Chi square test for categorical variables

**Table 4:** Comparison of early pregnancy factors for GDM in overweight/obese women (PPBMI ≥ 25 kg/m^2^) with and without PCOS

	PCOS (n = 95)	Non-PCOS (n = 139)	p-value*
Gravidity, mean ± SD	1.70 ± 0.97	1.96 ± 1.09	0.07
Parity, mean ± SD	1.06 ± 1.01	1.05 ± 1.01	0.7
History of EPL, n (%)	19 (20.0)	34 (24.5)	0.4
IVF–ET, n (%)	10 (10.5)	4 (2.9)	*0.02*
Family history of DM, n (%)	26 (27.4)	28 (20.1)	0.2
Family history of hypertension, n (%)	35 (36.8)	49 (35.3)	0.8
SBP, mmHg, mean ± SD	119.4 ± 10.9	122.3 ± 10.4	0.05
DBP, mmHg, mean ± SD	73.84 ± 10.12	77.01 ± 8.62	*0.01*
FPG, mmol/L, mean ± SD	4.96 ± 0.48	5.01 ± 0.46	0.4
Fins, mIU/L, mean ± SD	21.43 ± 10.82	19.37 ± 9.72	0.2
Log^HoMA−IR^, mean ± SD	1.46 ± 0.47	1.34 ± 0.55	0.1
QUICKY, mean ± SD	0.31 ± 0.02	0.32 ± 0.03	0.1
Lipid profile			
TC, mmol/L, mean ± SD	4.62 ± 0.69	4.54 ± 0.77	0.4
TG, mmol/L, mean ± SD	1.87 ± 0.94	1.74 ± 1.20	0.4
HDL, mmol/L, mean ± SD	1.51 ± 0.37	1.50 ± 0.34	0.7
LDL, mmol/L, mean ± SD	2.20 ± 0.47	2.35 ± 0.66	0.05
ApoA1, mmol/L, mean ± SD	1.43 ± 0.41	1.33 ± 0.32	*0.046*
ApoB, mmol/L, mean ± SD	0.77 ± 0.15	0.79 ± 0.18	0.4
Lp (a), mg/L, mean ± SD	227.9 ± 267.1	289.1 ± 287.8	0.1
APN, μg/mL, mean ± SD	44.25 ± 22.83	49.79 ± 27.21	0.1
SHBG, nmol/L, mean ± SD	186.4 ± 103.7	190.3 ± 98.2	0.8

Italic values are statistically significant at p < 0.05

Family history of DM and hypertension means related history of first degree relatives

*GDM* gestational diabetes, *PPBMI* pre-pregnancy body mass index, *PCOS* polycystic ovary syndrome, *EPL* early pregnancy loss, *IVF–ET* in vitro fertilization and embryo transfer, *DM* diabetes mellitus, *SBP* systolic blood pressure, *DBP* diastolic blood pressure, *FPG* fasting plasma glucose, *Fins* fasting insulin, *HoMA-IR* homeostasis model assessment-insulin resistance, *QUICKI* Quantitative insulin sensitivity check index, *TC* total cholesterol, TG triglycerides, HDL high density lipoprotein-cholesterol, *LDL* low density lipoprotein-cholesterol, *ApoA1* apolipoprotein A-I, *ApoB* apolipoprotein B, *Lp (a)* lipoprotein a, *APN* adiponectin, *SHBG* sex hormone-binding globulin

* p-value was calculated by student t-test for numeric variables and by Chi square test for categorical variables

We further tested our findings in a separate and bigger cohort, which included pregnant women who delivered at our hospital from February, 2016 to December 2017. There are total 18,106 women in this cohort, 877 (4.8%) of which had a diagnosis of PCOS (Table 5). The overall GDM prevalence was 17.2% in this population. We analyzed this group after stratification by body weight and discovered that in women with BMI < 25 kg/m^2^, the rate of GDM is significantly higher in PCOS than in non-PCOS women (22.09% vs. 14.52%, p < 0.0001). Whereas, in overweight/obese women, PCOS does not appear to confer additional risk of GDM.

**Table 5:** Confirmation of risk for GDM in women with and without PCOS stratified by PPBMI in a separate cohort (n = 18106)

	PCOS (n = 877)	Non-PCOS (n = 17,229)	p-value*
Age, years, mean ± SD	31.04 ± 3.44	31.46 ± 4.03	*0.0002*
Gravidity, mean ± SD	1.73 ± 0.96	1.93 ± 1.10	< *0.0001*
PPBMI, kg/m^2^, mean ± SD	23.52 ± 3.72	21.67 ± 3.11	*0.0002*
Prevalence of overweight/obesity	266 (30.33)	2265 (13.15)	< *0.0001*
Family history of DM, n (%)	164 (18.70)	2057 (11.94)	< *0.0001*
Family history of hypertension, n (%)	267 (30.44)	3415 (19.82)	< *0.0001*
GDM, n (%)			
< 25 kg/m^2^	135 (22.09)	2173 (14.52)	< *0.0001*
≥25 kg/m^2^	88 (33.08)	718 (31.70)	0.65
Total	223 (25.43)	2891 (16.78)	< *0.0001*

Italic values are statistically significant at p < 0.05

Family history of DM and hypertension means related history of first degree relatives

*GDM* gestational diabetes, *PCOS* polycystic ovary syndrome, *PPBMI* pre-pregnancy body mass index, *DM* diabetes mellitus, *SBP* systolic blood pressure

* p-value was calculated by student t-test for numeric variables and by Chi square test for categorical variables

## Discussion

This study demonstrated that early pregnancy metabolic factors and risk of GDM in PCOS women varied in different pre-pregnancy body weight groups. In normal weight women, PCOS is associated with higher prevalence of GDM, a trend towards higher insulin resistance, higher TG and lower SHBG levels. In contrast, in the overweight/obese group, GDM prevalence and risk factors for GDM appear to be independent of PCOS. By conducing analyses in a different but larger cohort, we are able to confirm that PCOS-related risk of GDM differed in normal weight and overweight/obese groups.

Previous studies in different ethnic groups have consistently shown an increased risk of GDM in PCOS women [5, 9, 14]. However, the exact mechanisms underlying this enhanced risk of GDM in PCOS is not clearly understood. Approximately half of women with PCOS are overweight or obese and excessive adiposity is a well-known risk factor for GDM [15]. However, whether obesity is responsible for the increased risk of GDM in women with PCOS is a continuing matter of debate [6]. A study by Mustaniemi et al. indicated that risk of GDM in women with PCOS is mainly related to adiposity [16]. Another meta-analyses suggested that PCOS itself was associated with a significant increased risk of GDM [5]. A study by Ollila et al. suggested that PCOS per se significantly increased the risk of type 2 diabetes in overweight/obese women [4]. Even less is known regarding the pathogenesis of GDM in normal weight PCOS women.

Our study revealed that the increased risk of GDM in PCOS women varied in different weight groups, in which the higher prevalence of GDM was only observed in normal weight but not in overweight/obese PCOS women. There are differences in risk for GDM in overweight/obese PCOS women between this study and previous reports, possibly due to ethnic variations. Anyhow, Overweight/obese women, regardless of PCOS status, are usually considered high risk group for metabolic disorders during pregnancy and thus normally receive close monitoring and intervention during their prenatal care. Our findings highlight that normal weight women with PCOS should also deserve close monitoring for hyperglycemia given their increased risk for GDM compared to normal weight women without PCOS.

In addition, our study suggested that normal weight women with PCOS showed higher TG, lower SHBG levels, and a trend towards increased insulin resistance. We speculated that the presence of early-pregnancy metabolic risk factors may accounted for the increased risk of GDM. Insulin resistance has been consistently tied to the risk of GDM [17, 18], while TG level is closely related to insulin resistance [19, 20]. SHBG is the primary binding protein for sex hormones, which is involved in various physiological and pathological conditions. SHBG mainly regulates the level of free sex hormones and thereby play an important role in the development of PCOS [21, 22] by modulating the biologic effects of sex hormones on peripheral tissues [23, 24]. Sex hormones bound to SHBG may amplify cell-surface signaling, thereby affect expression of insulin signaling pathway components [23–25]. The difference in early pregnancy metabolic factors between normal weight women with and without PCOS suggested that the pathophysiology of GDM might differ in this specific population, as compared to overweight/obese women in whom the risk of GDM seems to be independent of PCOS.

However, the lack of a difference in risk of GDM between normal weight and overweight/obese women with/without PCOS in the initial cohort may be due to a statistical type II error, i.e., failing to reject a false null hypothesis. In a confirmative cohort with a larger sample size, we were able to observe significant difference between these groups. In addition, the history of successful pregnancy and the prevalence of GDM were not reported in this study. The most obese women with presence of insulin resistance are less likely to fall pregnant compared to women with lower BMI. There are also limitations of HOMA in assessing insulin resistance. Furthermore, stratified analyses by BMI may produce residual confounding. Another limitation of the study is that early pregnancy factors were measured up to 15 weeks of gestation in some participants.

It is well known that GDM is highly associated with a future risk for Type 2 diabetes [26]. It would be interesting to know whether normal weight PCOS women would have an increased risk for Type 2 diabetes after having GDM during pregnancy. A recent study demonstrated an increased risk of Type 2 diabetes only in overweight and obese, but not in normal weight women with PCOS [4]. More studies are needed to ascertain the future risk of Type 2 diabetes in normal weight PCOS women who developed GDM.

Previous studies has reported increased risk of perinatal outcomes in women with PCOS, including PIH, pre-eclampsia, preterm birth, and perinatal mortality [7, 14, 27, 28]. However, in this study, there is no difference in pregnancy outcomes between women with and without PCOS except for the risk of GDM.

In conclusion, PCOS is associated with higher risk of GDM in normal weight women, while the risk of GDM is independent of PCOS in overweight and obese women. Normal weight women with PCOS had higher TG and lower SHBG levels, and a possible trend towards higher insulin resistant during early pregnancy compared to normal weight women without PCOS. These findings suggest that normal weight women with PCOS should be considered high risk of GDM.

## Data Availability

The data is available upon reasonable request to the corresponding author.

## References

[1] Broekmans FJ, Knauff EA, Valkenburg O, Laven JS, Eijkemans MJ, Fauser BC (2006). PCOS according to the Rotterdam consensus criteria: change in prevalence among WHO-II anovulation and association with metabolic factors. BJOG.

[2] Bellver J, Rodriguez-Tabernero L, Robles A, Munoz E, Martinez F, Landeras J, Garcia-Velasco J, Fontes J, Alvarez M, Alvarez C (2018). Polycystic ovary syndrome throughout a woman’s life. J Assist Reprod Genet.

[3] Teede HJ, Hutchison S, Zoungas S, Meyer C (2006). Insulin resistance, the metabolic syndrome, diabetes, and cardiovascular disease risk in women with PCOS. Endocrine.

[4] Ollila MM, West S, Keinanen-Kiukaaniemi S, Jokelainen J, Auvinen J, Puukka K, Ruokonen A, Jarvelin MR, Tapanainen JS, Franks S (2017). Overweight and obese but not normal weight women with PCOS are at increased risk of Type 2 diabetes mellitus-a prospective population-based cohort study. Hum Reprod.

[5] Boomsma CM, Eijkemans MJ, Hughes EG, Visser GH, Fauser BC, Macklon NS (2006). A meta-analysis of pregnancy outcomes in women with polycystic ovary syndrome. Hum Reprod Update.

[6] Boomsma CM, Fauser BC, Macklon NS (2008). Pregnancy complications in women with polycystic ovary syndrome. Sem Reprod Med.

[7] Sawada M, Masuyama H, Hayata K, Kamada Y, Nakamura K, Hiramatsu Y (2015). Pregnancy complications and glucose intolerance in women with polycystic ovary syndrome. Endocr J.

[8] Palomba S, La Sala GB (2016). Pregnancy complications in women with polycystic ovary syndrome: importance of diagnostic criteria or of phenotypic features?. Hum Reprod.

[9] Yu HF, Chen HS, Rao DP, Gong J (2016). Association between polycystic ovary syndrome and the risk of pregnancy complications: a PRISMA-compliant systematic review and meta-analysis. Medicine.

[10] Yilmaz M, Biri A, Bukan N, Karakoc A, Sancak B, Toruner F, Pasaoglu H (2005). Levels of lipoprotein and homocysteine in non-obese and obese patients with polycystic ovary syndrome. Gynecol Endocrinol.

[11] Goodarzi MO, Dumesic DA, Chazenbalk G, Azziz R (2011). Polycystic ovary syndrome: etiology, pathogenesis and diagnosis. Nat Rev Endocrinol.

[12] Arulkumaran N, Lightstone L (2013). Severe pre-eclampsia and hypertensive crises. Best Pract Res Clin Obstetr Gynaecol.

[13] Villar J, Cheikh Ismail L, Victora CG, Ohuma EO, Bertino E, Altman DG, Lambert A, Papageorghiou AT, Carvalho M, Jaffer YA (2014). International standards for newborn weight, length, and head circumference by gestational age and sex: the Newborn Cross-Sectional Study of the INTERGROWTH-21st Project. Lancet.

[14] Palomba S, de Wilde MA, Falbo A, Koster MP, La Sala GB, Fauser BC (2015). Pregnancy complications in women with polycystic ovary syndrome. Hum Reprod Update.

[15] Norman RJ, Noakes M, Wu R, Davies MJ, Moran L, Wang JX (2004). Improving reproductive performance in overweight/obese women with effective weight management. Hum Reprod Update.

[16] Mustaniemi S, Vaarasmaki M, Eriksson JG, Gissler M, Laivuori H, Ijas H, Bloigu A, Kajantie E, Morin-Papunen L (2018). Polycystic ovary syndrome and risk factors for gestational diabetes. Endocr Connect.

[17] Rodrigo N, Glastras SJ (2018). The emerging role of biomarkers in the diagnosis of gestational diabetes mellitus. J Clin Med.

[18] Kuhl C (1991). Insulin secretion and insulin resistance in pregnancy and GDM. Implications for diagnosis and management. Diabetes.

[19] Kraegen EW, Cooney GJ, Ye J, Thompson AL (2001). Triglycerides, fatty acids and insulin resistance–hyperinsulinemia. Exp Clin Endocrinol Diabetes..

[20] Gonzalez-Chavez A, Simental-Mendia LE, Elizondo-Argueta S (2011). Elevated triglycerides/HDL-cholesterol ratio associated with insulin resistance. Cirugia y cirujanos.

[21] Martinez-Garcia MA, Gambineri A, Alpanes M, Sanchon R, Pasquali R, Escobar-Morreale HF (2012). Common variants in the sex hormone-binding globulin gene (SHBG) and polycystic ovary syndrome (PCOS) in Mediterranean women. Hum Reprod.

[22] Veltman-Verhulst SM, van Haeften TW, Eijkemans MJ, de Valk HW, Fauser BC, Goverde AJ (2010). Sex hormone-binding globulin concentrations before conception as a predictor for gestational diabetes in women with polycystic ovary syndrome. Hum Reprod.

[23] Nestler JE (2009). Sex hormone-binding globulin and risk of type 2 diabetes. N Engl J Med.

[24] Tawfeek MA, Alfadhli EM, Alayoubi AM, El-Beshbishy HA, Habib FA (2017). Sex hormone binding globulin as a valuable biochemical marker in predicting gestational diabetes mellitus. BMC Women’s Health.

[25] Zhang B, Jin Z, Sun L, Zheng Y, Jiang J, Feng C, Wang Y (2016). Expression and correlation of sex hormone-binding globulin and insulin signal transduction and glucose transporter proteins in gestational diabetes mellitus placental tissue. Diabetes Res Clin Pract.

[26] Bellamy L, Casas JP, Hingorani AD, Williams D (2009). Type 2 diabetes mellitus after gestational diabetes: a systematic review and meta-analysis. Lancet.

[27] Kollmann M, Klaritsch P, Martins WP, Guenther F, Schneider V, Herzog SA, Craciunas L, Lang U, Obermayer-Pietsch B, Lerchbaum E (2015). Maternal and neonatal outcomes in pregnant women with PCOS: comparison of different diagnostic definitions. Hum Reprod.

[28] Klevedal C, Turkmen S (2017). Fetal–maternal outcomes and complications in pregnant women with polycystic ovary syndrome. Minerva Ginecol.

